# Mitochondrial and ribosomal biogenesis are new hallmarks of stemness, oncometabolism and biomass accumulation in cancer: Mito‐stemness and ribo‐stemness features

**DOI:** 10.18632/aging.102054

**Published:** 2019-07-16

**Authors:** Maria Peiris‐Pagès, Béla Ozsvári, Federica Sotgia, Michael P. Lisanti

**Affiliations:** 1 Clinical and Experimental Pharmacology, University of Manchester, Cancer Research UK, Manchester, United Kingdom; 2 Translational Medicine, School of Environment and Life Sciences, Biomedical Research Centre (BRC), University of Salford, Greater Manchester, United Kingdom

**Keywords:** coculture, tumor microenvironment, proteomics, mito-stemness, ribo-stemness, mitochondria, protein synthesis, organelle biogenesis

## Abstract

Using proteomics analysis, we previously compared MCF7 breast cancer cells grown as 3D tumor spheres, with the same cell line grown as monolayers. Our results indicated that during 3D anchorage‐independent growth, the cellular machinery associated with i) mitochondrial biogenesis and ii) ribosomal biogenesis, were both significantly increased. Here, for simplicity, we refer to these two new oncogenic hallmarks as “mito‐stemness” and “ribo‐stemness” features. We have now applied this same type of strategy to begin to understand how fibroblasts and MCF7 breast cancer cells change their molecular phenotype, when they are co‐cultured together. We have previously shown that MCF7‐fibroblast co‐cultures are a valuable model of resistance to apoptosis induced by hormonal therapies, such as Tamoxifen and Fulvestrant. Here, we directly show that these mixed co‐cultures demonstrate the induction of mito‐stemness and ribo‐stemness features, likely reflecting a mechanism for cancer cells to increase their capacity for accumulating biomass. In accordance with the onset of a stem‐like phenotype, KRT19 (keratin 19) was induced by ~6‐fold during co‐culture. KRT19 is a well‐established epithelial CSC marker that is used clinically to identify metastatic breast cancer cells in sentinel lymph node biopsies. The potential molecular therapeutic targets that we identified by label‐free proteomics of MCF7‐fibroblast co‐cultures were then independently validated using a bioinformatics approach. More specifically, we employed publically‐available transcriptional profiling data derived from primary tumor samples from breast cancer patients, which were previously subjected to laser‐capture micro‐dissection, to physically separate breast cancer cells from adjacent tumor stroma. This allowed us to directly validate that the proteins up‐regulated in this co‐culture model were also transcriptionally elevated in patient‐derived breast cancer cells *in vivo*. This powerful approach for target identification and translational validation, including the use of patient outcome data, can now be applied to other tumor types as well, to validate new therapeutic targets that are more clinically relevant, for patient benefit. Moreover, we discuss the therapeutic implications of these findings for new drug development, drug repurposing and Tamoxifen‐resistance, to effectively target mito‐stemness and ribo‐stemness features in breast cancer patients. We also discuss the broad implications of this “organelle biogenesis” approach to cancer therapy.

## Introduction

Cancer stem cells (CSCs) are now thought to be one of the major drivers behind treatment failure in many cancer types, including breast cancer [1]. As a con- sequence, residual CSCs, which are chemo-resistant and radio-resistant, result in tumor recurrence and distant metastasis, ultimately killing most cancer patients [2]. Therefore, there is an urgent need to understand what are the metabolic weak points within CSCs, to drive new drug discovery, for patient benefit. This requires new innovative approaches towards understanding “stemness” features and identifying specific metabolic targets in CSCs [3–5].

In order to identify new characteristic features of “stemness” in cancer cells, we previously compared the proteomic profiles of 3D-spheroid cultures of MCF7 breast cancer cells, with MCF7 monolayer cells, processed in parallel [6]. These 3D-spheroids were grown under anchorage-independent conditions and are also known as mammosphere cultures, which are highly enriched in CSCs and cancer progenitor cells [6]. Using this approach, we previously demonstrated that under these 3D growth conditions, MCF7 cells up-regulated the expression of >60 mitochondrial-related proteins and >80 proteins related to protein synthesis, including ribosomal biogenesis [6,7]. Moreover, we have shown that pharmacologically targeting protein synthesis and/or mitochondrial function are both indeed sufficient to eradicate CSCs [8–14].

Here, we refer to these characteristic proteomic changes as “mito-stemness” [15] and “ribo-stemness”. We have now investigated whether these stemness features are also similarly up-regulated during the co- culture of MCF7 breast cancer cells with fibroblasts. Our current observations are consistent with the idea that MCF7-fibroblast co-cultures increase their biosynthetic cellular machinery, to expand their capacity to increase biomass. Therefore, “mito- stemness” and “ribo-stemness” features are actually oncogenic hallmarks of the ability and readiness of cancer cells to aggressively undergo biomass accumulation.

We also discuss the implications of our current findings for understanding and treating Tamoxifen-resistance, as we have previously validated that the MCF7-fibroblast co-culture system is a bonafide model of Tamoxifen- resistance [16], which includes a stromal micro- environment, making it perhaps more physiologically- relevant.

## RESULTS

### Unbiased proteomics analysis: identification of proteins up-regulated in MCF7-fibroblast co- cultures

Previously, we have demonstrated that cancer cells, grown in close proximity to fibroblasts, metabolically reprogram these fibroblasts towards a more catabolic state, via the induction of autophagy, mitophagy and glycolysis, as well as senescence [16–20]. Conversely, through this interaction, cancer cells undergo reciprocal metabolic reprogramming towards a more anabolic state, with the induction of mitochondrial biogenesis and oxidative metabolism [21,22]. This metabolic co-operation primarily benefits the cancer cells by providing nutrients to generate new biomass [17,18]. Unfortunately, most of the metabolic targets in this symbiotic process remain completely un- known.

**Figure 1:**
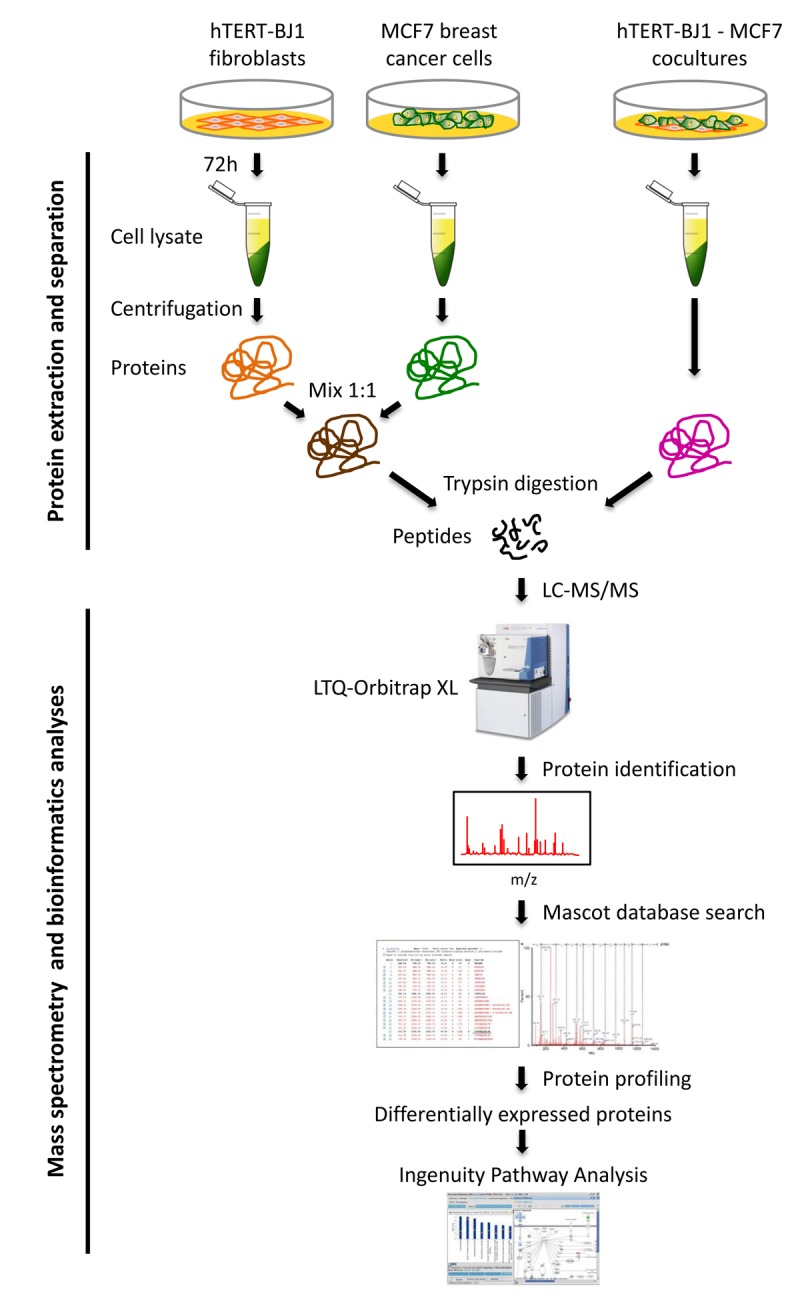
**Schematic diagram summarizing the work‐flow for MCF7‐fibroblast co‐culture studies and bioinformatics validation.** Protein lysates were obtained from hTERT‐BJ1 fibroblasts after 72 h co‐culture with MCF7 breast cancer cells. Alternatively, protein lysates were obtained from hTERT‐BJ1 fibroblasts and MCF7 cells cultured separately as monolayers and then mixed. Peptides obtained after trypsin digestion were analysed via LC‐MS/MS on an LTQ‐ Orbitrap XL mass spectrometer. Label‐free quantitative proteomics was used to detect changes in protein abundances across co‐cultures and mixed cell population extracts. The proteomics data sets were further analyzed using Ingenuity Pathway Analysis. This co‐culture approach is predicted to better simulate the fibroblast‐rich local tumor micro‐environment *in vivo*.

Here, we developed a new approach to identify these potential therapeutic targets, via unbiased proteomics analysis. For this purpose, we used MCF7 cells, an ER(+) human breast cancer cell line, as a model system. These MCF7 cells were co-cultured with hTERT-BJ1 fibroblasts, in a cellular ratio of 1:1, for a short 3-day period. These MCF7-fibroblast co-cultures were then directly compared to a 1:1 protein mixture of MCF7 cells and hTERT-fibroblasts, that had not been co- cultured together, but were grown instead as homotypic mono-cultures (Figure 1).

**Figure 2:**
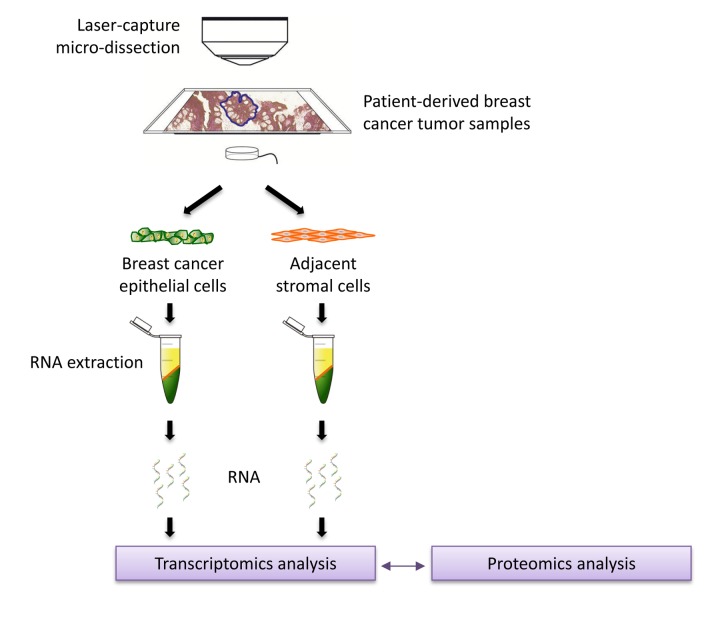
**Schematic diagram summarizing the work‐flow for validation studies with transcriptional profiling data from clinical samples.** Data from N=28 patient‐derived breast primary tumor samples, which have been subjected to laser‐capture micro‐dissection, were used for validation.

To a first approximation, using proteomics, this approach should allow us to estimate and identify which proteins are increased during the co-culture process, relative to mono-cultures. Then, these protein candidates were compared with human breast cancer samples that had undergone laser-capture micro- dissection, to validate their relevant expression in human breast cancer cells *in vivo*. For this purpose, we used publicly-available transcriptional-profiling data (from N=28 breast cancer patients; See Materials and Methods). A diagram highlighting this work-flow is shown as Figure 2.

Previously, we have shown that mitochondrial biogenesis in MCF7 cells is induced when they are co- cultured with fibroblasts [21,22]. However, it remains unknown exactly which mitochondrial proteins are induced during co-culture. Therefore, we focused first on which mitochondrial proteins were increased during co-culture, based on this proteomics approach.

Supplementary Table 1 illustrates the 45 mitochondrial- related proteins that were found to be significantly up- regulated during the MCF7-fibroblast co-culture process. This list includes proteins that are involved in mitochondrial biogenesis and/or are part of the mitochondrial complexes I to V, as well as mitochondrial chaperones, such as DNAJA3, HSPD1 and HSPA9, among others. Interestingly, NDUFAF2, a MYC-induced component of complex I [23], was infinitely up-regulated during co-culture. Interestingly, we have previously shown that the co-culture of MCF7 cells with fibroblasts confers Tamoxifen-resistance, that is reversible by treatment with Metformin, a complex I inhibitor [16]. Therefore, NDUFAF2 (a.k.a, Mimitin [23]) may be functionally conferring Tamoxifen- resistance during co-culture.

Since increased mitochondrial biogenesis is a hallmark of cancer stem cells (CSCs), known as “mito-stemess” [6–8,15], we next also looked for markers of protein synthesis [7], that is another hallmark of CSCs, which we now refer to here as “ribo-stemness”. Supplementary Table 2 shows that 28 components of the large and small cellular ribosomal subunits were up-regulated during co-culture. More specifically, 18 components of the large 60S ribosome and 10 components of the small 40S ribosome were increased. Interestingly, RPL4 and RPS29 were infinitely increased during co-culture.

Consistent with these findings, protein-folding chaperones were also induced. Supplementary Table 3 lists 10 chaperones that were significantly increased during co-culture. These include members of the HSP90 and HSP70 families of chaperones, as well as others. Remarkably, HSP90AB1 was infinitely up-regulated.

Similarly, Supplementary Table 4 shows that proteins involved in mRNA translation initiation, polypeptide elongation, tRNA synthesis and amino acid uptake were all significantly up-regulated during co-culture. Overall, this includes 30 proteins in total. For example, EIF2S1, a translation initiation factor required for mRNA binding to ribosomes, was increased by nearly 300-fold.

Furthermore, other well-known markers of “stemness” and proliferation were increased, as shown in Supplementary Table 5. More specifically, MKI67 was increased by >4,000-fold, while KRT19 and PCNA were increased by nearly 6-fold and 4-fold, respectively. The profound increase in MKI67 is more consistent with increased protein synthesis, rather than increased proliferation. Interestingly, MKI67 is expressed in all cycling cells, except for resting cells in the G0-phase and is specifically associated with ribosomal RNA (rRNA) synthesis and, thus, protein synthesis. This is consistent with our results presented in Supplementary Tables 2-4.

In this context, it is interesting to note that KRT19 is a well-established epithelial CSC marker that is used clinically to identify metastatic breast cancer cells in sentinel lymph node biopsies [24,25].

A summary of the proteins that were up-regulated by >100-fold is highlighted in Table 1. In conclusion, our current observations are consistent with the idea that MCF7-fibroblast co-cultures increase their biosynthetic cellular machinery (i.e., mitochondria and ribosomes), to expand their anabolic capacity to increase their biomass.

**Table 1:** Proteomics summary: key targets that were up-regulated by >100-fold in MCF7-fibroblast co-cultures.

**Gene**	**Description**	**Fold-increase**
		
**1. Mitochondrial Proteins**		
**NDUFAF2**	**Mimitin, c-Myc-induced mitochondrial protein; B17.2L**	**Infinity**
**GPD2**	**Glycerol-3-phosphate dehydrogenase, mitochondrial**	**238.91**
**AIFM1**	**Apoptosis-inducing factor 1, mitochondrial**	**237.74**
**PRKDC**	**DNA-dependent protein kinase catalytic subunit**	**102.78**
		
**2. Ribosomal Proteins**		
**RPL4**	**60S ribosomal protein L4**	**Infinity**
**RPS29**	**40S ribosomal protein S29**	**Infinity**
**RPL15**	**60S ribosomal protein L15**	**2,238.12**
**RPL19**	**60S ribosomal protein L19**	**168.84**
		
**3. Chaperone and Translation** **initiation factors**		
**HSP90AB1**	**Heat Shock Protein 90kDa Alpha (Cytosolic), Class B Member 1**	**Infinity**
**EIF2S1**	**Eukaryotic translation initiation factor 2 subunit 1**	**291.47**
		
**4. Ribosomal RNA (rRNA) synthesis**		
**MKI67**	**Antigen KI-67**	**4,531.38**

### Ingenuity pathway analysis (IPA) of MCF7- fibroblast co-cultures

Differentially expressed proteins were also independently analyzed using Ingenuity Pathway Analysis (IPA), to identify altered canonical pathways and toxicity functions.

**Figure 3:**
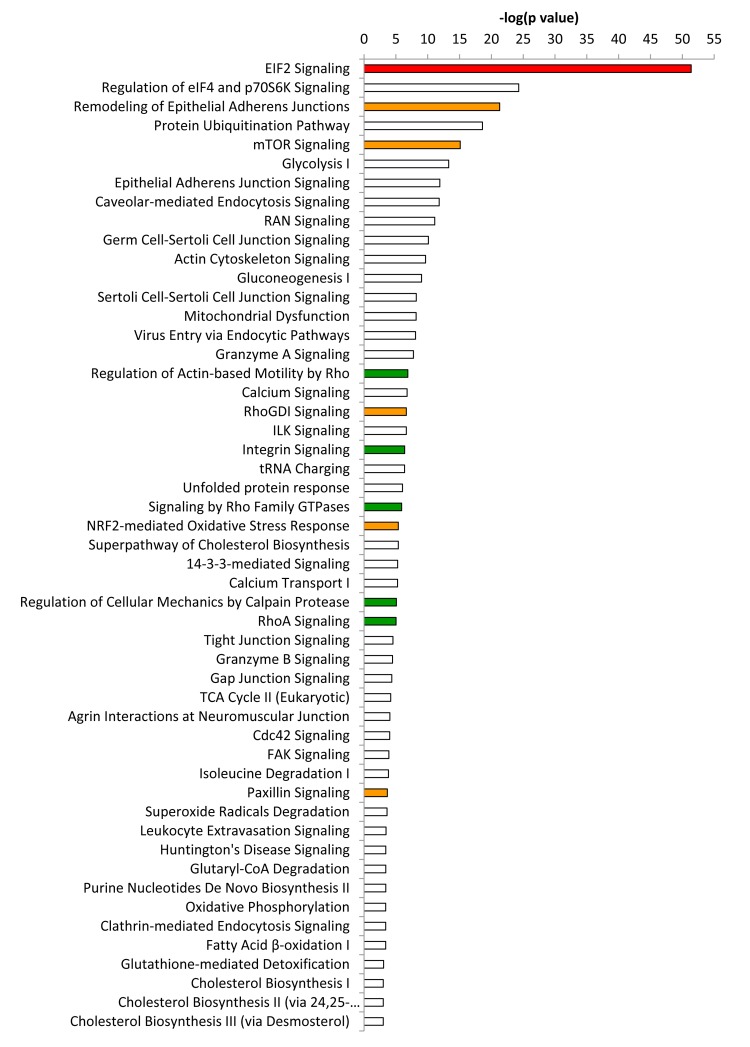
**Ingenuity pathway analysis of differentially expressed proteins in co‐cultures compared to mixed cell populations.** Ingenuity Pathway Analysis (IPA) showed canonical pathways significantly altered (p < 0.001). The p value for each pathway is indicated by the bar and is expressed as ‐1 times the log of the p value. Red colored bars indicate a predicted significant activation of the pathway (z‐score >2), whereas orange colored bars indicate a not significant activation (z‐score between 0 and 2). **Green** bars indicate a not significant inhibition of the pathway (z‐score between 0 and ‐2). White bars indicate that the pathway is altered, but it was not possible to predict whether it is activated nor inhibited.

**Figure 4:**
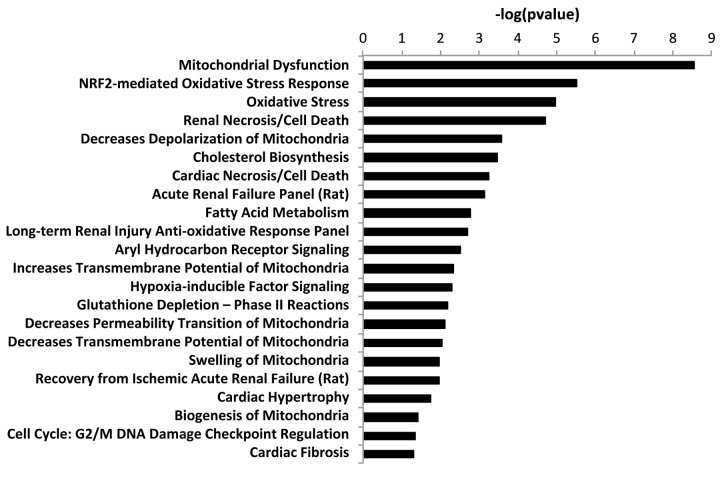
**“Toxicity” effects of differentially expressed proteins in co‐cultures versus monocultures.** Ingenuity Pathway Analysis showed toxicity functions significantly enriched by the proteins differentially expressed in co‐cultures (p < 0.05). The p value for each pathway is indicated by the bar and is expressed as ‐1 times the log of the p value.

This analysis revealed that EIF2 signaling, which is crucial for protein synthesis, is a significantly activated canonical pathway in co-cultures, as compared to mono- cultures, as measured by a z-score > 2 (Figure 3). Other altered pathways and toxicity functions identified by IPA included mitochondrial dysfunction, NRF2- mediated oxidative stress, fatty acid metabolism, changes in mitochondrial membrane potential, HIF signaling, glutathione depletion, mitochondrial biogenesis, DNA-damage and fibrosis, among others (Figure 4). The observed similarity with renal injury appears to be related to an increase in oxidative stress and the resulting anti-oxidant response.

Overall, these results are consistent with the induction of “mito-stemness” and “ribo-stemness” features, during the co-culture process.

### Validating the clinical relevance of co-culture proteomics data, using patient-derived breast tumor samples

In order to directly validate the potential clinical relevance of our findings, we next used transcriptional profiling data derived from the analysis of N=28 breast cancer patients, which were previously subjected to laser-capture micro-dissection, to physically separate breast cancer cells from adjacent stromal cells [26].

Therefore, we intersected our proteomics data, with this clinical data set, and the levels of fold-upregulation observed in the epithelial cancer cell compartment (relative to the tumor stroma), and corresponding p- values derived from these clinical samples, are shown in Supplementary Tables 6 to 9.

Supplementary Table 6 shows that of the 45 mitochondrial proteins that were up-regulated during co-culture, 34 were also significantly increased by >1.75-fold in human breast cancer cells *in vivo*. Therefore, >75% of the mitochondrial proteins elevated during co-culture were transcriptionally increased in patient-derived breast cancer cells *in vivo*.

Similarly, Supplementary Table 7 highlights that of the 28 ribosomal proteins that were up-regulated during co- culture, 27 were significantly increased by >1.7-fold in human breast cancer cells *in vivo*. Thus, >96% of the ribosomal proteins elevated during co-culture were transcriptionally increased in human breast cancer cells *in vivo*.

In addition, Supplementary Table 8 illustrates that of the 10 chaperone proteins that were up-regulated during co-culture, 7 were significantly increased by >3.1-fold in human breast cancer cells *in vivo*. As such, 70% of the chaperone proteins increased during co-culture were also increased in human breast cancer cells.

Finally, Supplementary Table 9 shows that of the 30 proteins associated with mRNA translation initiation, polypeptide elongation, and tRNA synthesis, 19 were significantly increased by >1.7-fold in human breast cancer cells *in vivo*. As a result, >60% of these proteins were also increased in human breast cancer cells.

Such a high concordance rate, between i) in vitro proteomics data and ii) in vivo human breast cancer transcriptional mRNA data, clearly validates the translational significance of the MCF7-fibroblast co- culture system, as a model for studying human breast cancer.

### Establishing new prognostic biomarkers and companion diagnostics for predicting tamoxifen- resistance, by exploiting proteomics data from MCF7-fibroblast co-cultures

**Figure 5:**
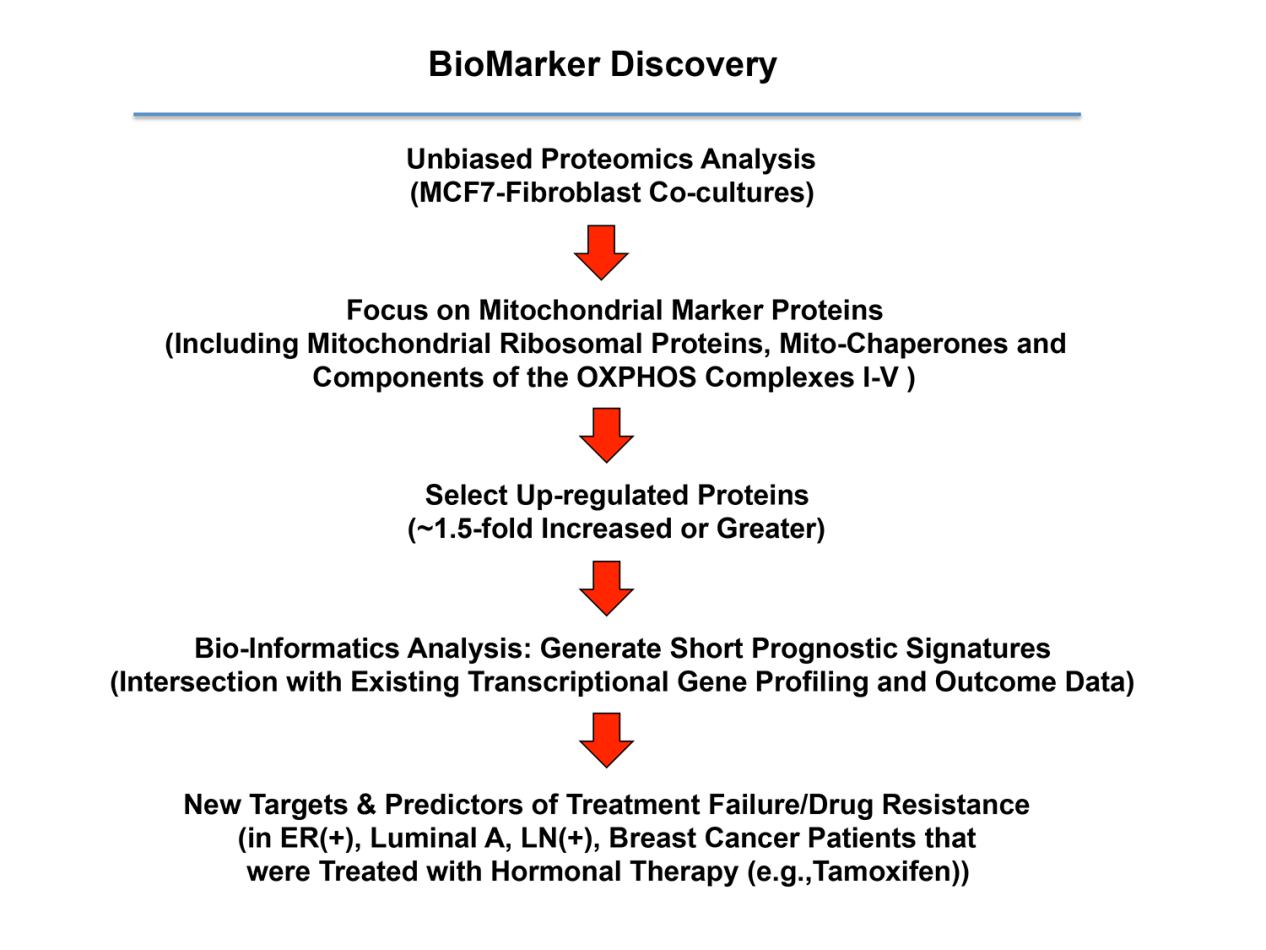
**A proteomics‐based approach to the development of new breast cancer companion diagnostics, for predicting Tamoxifen‐resistance.** For this analysis, data from the proteomics analysis of MCF7‐ fibroblast co‐cultures was intersected with clinical outcome data. More specifically, the clinical population we focused included ER(+) patients, of the luminal A sub‐type, that were lymph‐node positive (LN(+)) at diagnosis, who were treated with Tamoxifen and followed over nearly 200 months. In this context, we ultimately evaluated the prognostic value of a mitochondrial signature for predicting tumor treatment failure (recurrence, metastasis or overall survival).

We have previously shown that the co-culture of MCF7 cells with fibroblasts induces a Tamoxifen-resistance phenotype [16]. Therefore, to identify new potential biomarkers of Tamoxifen-resistance, here we intersected our proteomics data from MCF7-fibroblast co-cultures with publicly available transcriptional profiling data from the tumors of breast cancer patients that were treated with Tamoxifen, but did not receive any chemotherapy [27,28]. A schematic diagram summarizing this approach is shown in Figure 5.

For this purpose, we selected high-risk patients that were lymph-node positive at diagnosis, and we focused on the luminal A subtype, which represents the most common form of ER(+) breast cancers (N = 152 patients). The results of this analysis are shown in Table 2. In this context, seven distinct mitochondrial genes, represented by 9 different gene probes, showed significant prognostic value, and were able to predict tumor recurrence.

**Table 2:** Prognostic value of mitochondrial markers induced during metabolic symbiosis (MCF7-fibro) for tumor recurrence.

**Gene Probe ID**	**Symbol**	**Hazard-Ratio (RFS)**	**Log-Rank Test**
			
**211662_s_at**	**VDAC2**	**3.96**	**6.70E-07**
**200807_s_at**	**HSPD1**	**3.46**	**1.30E-05**
**200806_s_at**	**HSPD1**	**2.34**	**0.005**
**203633_at**	**CPT1A**	**2.86**	**0.01**
**203634_s_at**	**CPT1A**	**2.32**	**0.025**
**202698_x_at**	**COX4I1**	**2.19**	**0.038**
**211971_s_at**	**LRPPRC**	**2.05**	**0.01**
**200657_at**	**SLC25A5**	**2.4**	**0.002**
**221235_s_at**	**TRAP1**	**1.77**	**0.048**

RFS: recurrence-free survival.

**Figure 6:**
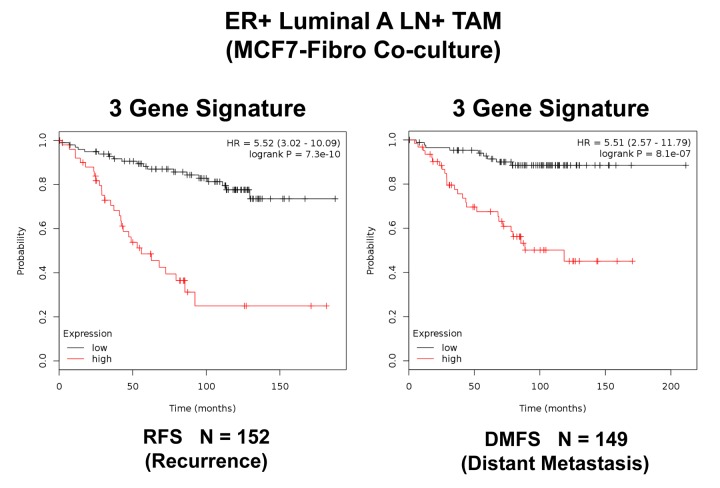
**A three‐gene based mitochondrial signature (Mito‐Signature) that effectively predicts recurrence and distant metastasis in high‐risk ER(+) breast cancer patients.** Note that this Mito‐Signature (HSPD1/VDAC2/CPT1A) predicts tumor recurrence (Left; N = 152 patients; p = 7.3e‐10) and distant metastasis (Right; N = 149 patients; p = 8.1e‐07) in LN(+) luminal A patients treated with Tamoxifen therapy, indicative of treatment failure and Tamoxifen‐resistance. Patients with high‐expression levels of the Mito‐Signature showed a >5‐fold increase in recurrence and distant metastasis, while being treated with hormonal therapy. See also Tables 3 and 4. RFS, reccurence‐free survival; DMFS, distant metastasis‐free survival.

**Figure 7:**
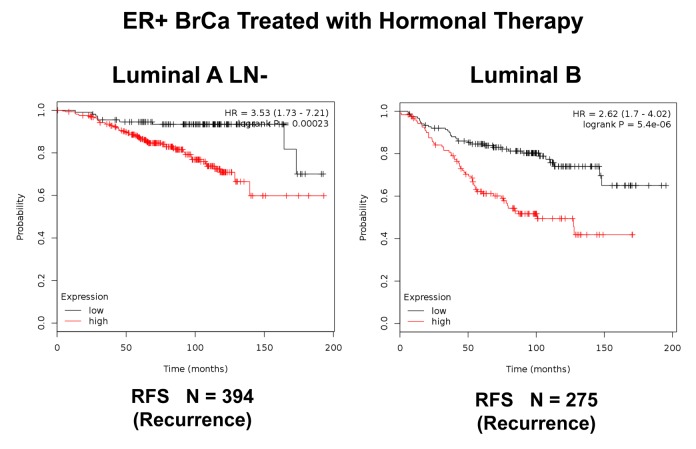
**K‐M analysis of a Mito‐Signature that shows predictive value in Luminal A (LN‐negative) and Luminal B breast cancer patients, who were treated with hormonal therapy.** Left, Luminal A/LN‐negative (N = 394 patients; p =0.00023). Right, Luminal B (N = 275 patients; p = 5.4e‐06). Patients with high‐expression levels of the Mito‐ Signature showed a clear increase in recurrence, while being treated with hormonal therapy. RFS, recurrence‐free survival.

**Figure 8:**
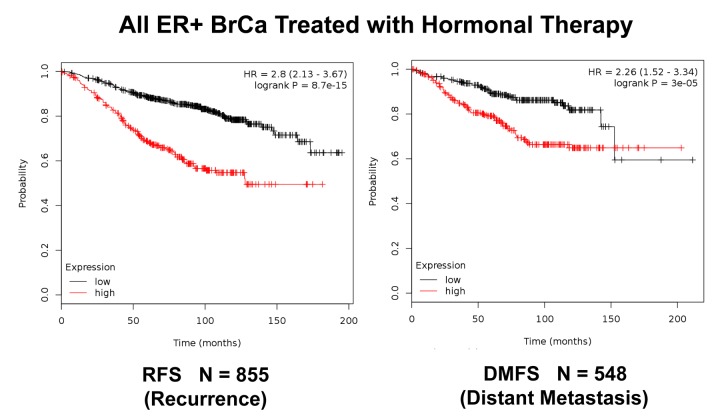
**K‐M analysis of recurrence and metastasis using a Mito‐Signature in a larger group of ER(+) breast cancer patients, who were treated with hormonal therapy.** These patients were not sub‐divided into luminal A/B subgroups and were not sub‐divided by lymph‐node status. Note that this Mito‐Signature effectively predicts tumor recurrence (Left; N = 855 patients) and distant metastasis (Right; N = 548 patients). Patients with high‐ expression levels of the Mito‐Signature showed a near 3‐fold increase in recurrence (p = 8.7e‐15) and a >2‐fold increase in distant metastasis (p = 3e‐05), while being treated with hormonal therapy.

**Figure 9:**
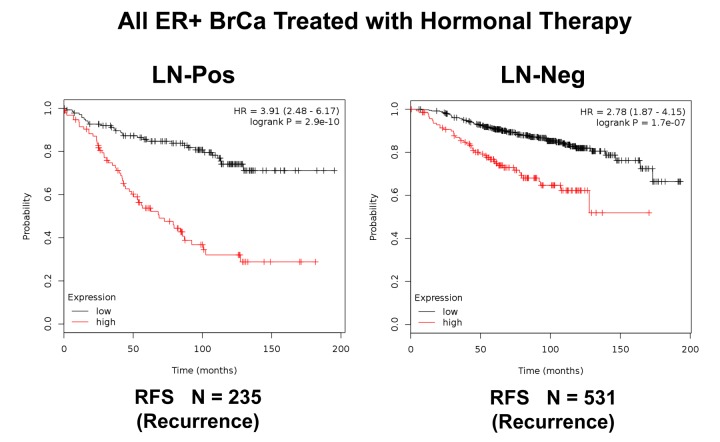
**K‐M analysis of recurrence using a Mito‐Signature in a larger group of ER(+) breast cancer patients, that were divided into sub‐groups by lymph node status, who were treated with hormonal therapy.** These patients were not sub‐divided into luminal A/B subgroups. Left, LN‐positive (N = 235 patients). Right, LN‐negative (N = 531 patients). Note that LN‐positive patients with high‐expression levels of the Mito‐Signature showed a near 4‐fold increase in recurrence, while being treated with hormonal therapy (p = 2.9 e‐10). Similar results were observed in LN‐negative patients, with a near 3‐fold increase in recurrence (p = 1.7e‐07). RFS, reccurence‐free survival.

**Figure 10:**
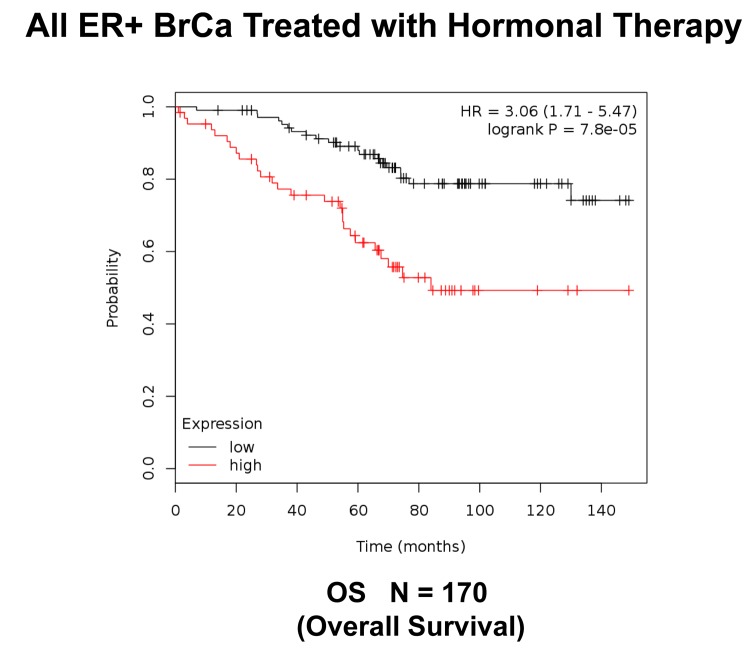
**K‐M analysis of survival using a Mito‐Signature in a group of ER(+) breast cancer.** These patients were treated with hormonal therapy. Note that patients with high‐expression levels of the Mito‐ Signature showed a >3‐fold reduction in long‐term survival (N = 170 patients; p = 7.8e‐05). OS, overall survival.

**Figure 11:**
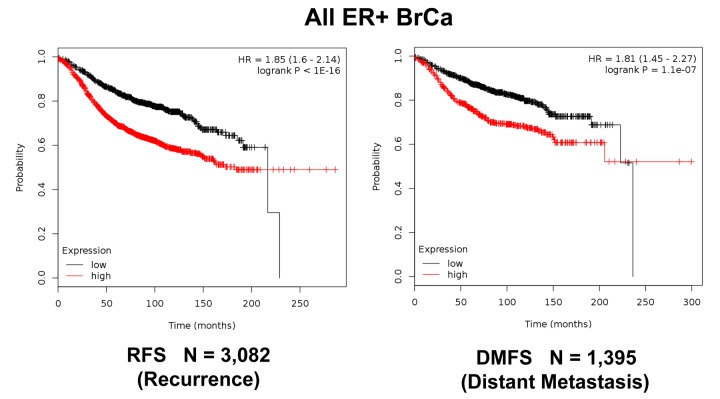
**K‐M analysis of recurrence and metastasis using a Mito‐Signature in a larger group of all ER(+) breast cancer patients, independently of treatment.** Patients with high‐expression levels of the Mito‐Signature showed near 2‐fold increases in recurrence (N = 3,082; p < 1e‐16) and distant metastasis (N = 1,395; p = 1.1e‐07).

**Figure 12:**
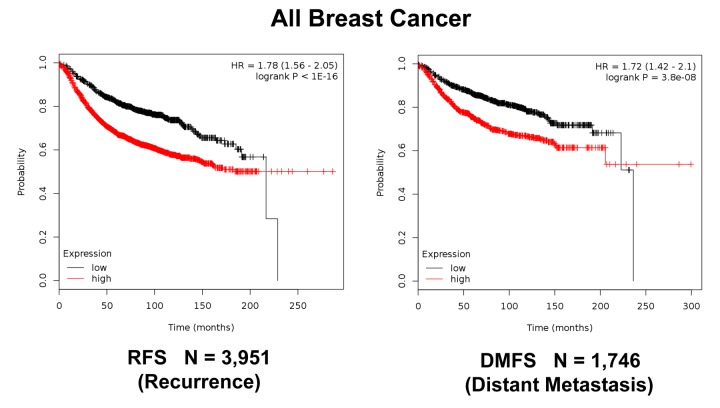
**K‐M analysis of recurrence and metastasis using a Mito‐Signature in a larger group of all breast cancer patients, independently of treatment.** Patients with high‐expression levels of the Mito‐Signature showed near 2‐fold increases in recurrence (N = 3,951; p < 1e‐16) and distant metastasis (N = 1,746; p = 3.8e‐08).

**Figure 13:**
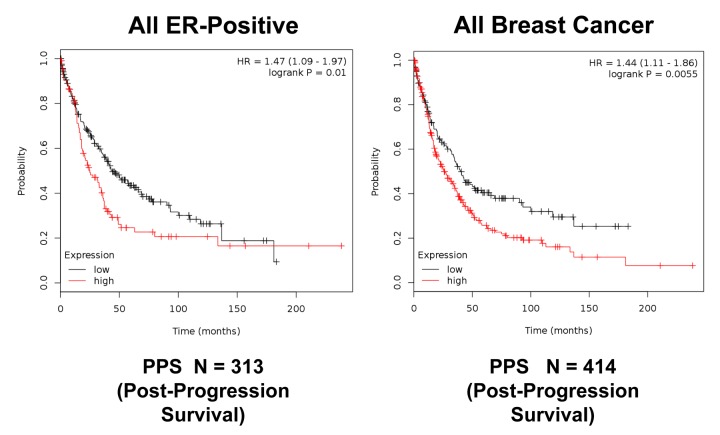
**K‐M analysis of post‐progression survival (PPS) using a Mito‐Signature in ER(+) and all breast cancer patients, independently of treatment.** Patients with high‐expression levels of the Mito‐Signature showed near 1.5‐fold reductions in post‐progression survival. Left, ER(+) (N = 313 patients; p = 0.01). Right, all breast cancer (N = 414 patients; p = 0.0055).

In order to increase the prognostic power of these individual mitochondrial biomarkers, we next selected the most promising ones and used them to create a new mitochondrial gene signature. This new Mito-Signature contains only 3 key genes (HSPD1, VDAC2, CPT1A) (Tables 3 and 4). K-M curves for this signature are shown in Figures 6 to 13.

**Table 3:** A 3-Gene mitochondrial signature for predicting treatment failure, due to tumor recurrence.

**Gene Probe ID**	**Symbol**	**Hazard-Ratio (RFS)**	**Log-Rank Test**
			
**211662_s_at**	**VDAC2**	**3.96**	**6.70E-07**
**200807_s_at**	**HSPD1**	**3.46**	**1.30E-05**
**203633_at**	**CPT1A**	**2.86**	**0.01**
**Combined**		**5.52**	**7.30E-10**

RFS: recurrence-free survival.

**Table 4:** A 3-Gene mitochondrial signature for predicting treatment failure, due to distant metastasis.

**Gene Probe ID**	**Symbol**	**Hazard-Ratio (DMFS)**	**Log-Rank Test**
			
**211662_s_at**	**VDAC2**	**3.11**	**0.0004**
**200807_s_at**	**HSPD1**	**3.5**	**9.70E-05**
**203633_at**	**CPT1A**	**2.79**	**0.026**
**Combined**		**5.51**	**8.10E-07**

DMFS: distant metastasis-free survival.

Importantly, this Mito-Signature yielded a significantly improved hazard-ratio for tumor recurrence of 5.52 (p = 7.3e-10). It was also highly predictive for distant metastasis, in the same group of patients (HR = 5.51; p = 8.1e-07). (See Figures 6). Similar results were obtained in Luminal A LN-negative patients and Luminal B patients (Figure 7).

We also examined the prognostic value of this mitochondrial gene signature in a larger group of ER(+) patients (N = 855), that received hormonal therapy, but not chemotherapy. This group of patients was not segregated into luminal A and luminal B subtypes. Figure 8 shows the results of this K-M analysis for relapse-free survival (HR = 2.80; p = 8.7e-15). Similar results were also obtained distant metastasis-free survival (HR = 2.26; p = 3e-05; N = 548 patients). This mitochondrial signature was also effective if the ER(+) patient population was divided into LN(+) and LN(-) groups (Figure 9).

Next, we assessed the behavior of this Mito-Signature in predicting overall survival. Figure 10 shows that it was also highly predictive of overall survival during hormonal therapy (HR = 3.06; p = 7.8e-05; N = 170 patients).

Moreover, Figure 11 shows that this Mito-Signature was also effective in all ER(+) breast cancer patients in predicting tumor recurrence (N = 3,082), as well as distant metastasis (N = 1,395). Similar results were obtained using data from all breast cancer patients, for recurrence (N = 3,951) and metastasis (N =1,746), as well as for post-progression survival (Figures 12 and 13). Thus, this mitochondrial-based gene signature may represent an important new prognostic tool for predicting patient outcomes, in a wide variety of different breast cancer patients, but especially in ER(+) patients treated with hormonal therapies.

### Markers of fibrosis and glycolysis are up-regulated in MCF7-fibroblast co-cultures, consistent with the onset of oxidative stress

During the co-culture of fibroblasts with cancer cells, it has been observed that their phenotype is drastically changed [17,18]. These changes are related to the induction of various biological processes related oxidative stress, and are consistent with a more myo- fibroblastic phenotype [17,18]. These phenotypic changes include increased expression of cytoskeletal elements and glycolytic enzymes, as well as the elevation of markers of autophagy and senescence [18,21,22]. Therefore, we examined our proteomics data for evidence of these biological processes.

Supplementary Table 10 illustrates that during MCF7- fibroblast co-cultures, many known markers of fibrosis and oxidative stress are actually increased. These changes include the up-regulation of 32 cytoskeletal and extracellular matrix proteins, 11 glycolytic enzymes, 4 lysosomal/autophagy markers and 2 markers of the senescence-associated secretory phenotype, known as SASP.

These findings are also consistent with our results from IPA analysis, shown in Figure 4. Therefore, our results potentially provide interesting new stromal targets for further validation in future studies.

### Validating the relevance of MCF7-fibroblast co- cultures for drug development, using FDA-approved antibiotics that target mitochondria

Based on our current findings, we observed that 45 mitochondrial proteins were significantly increased during the co-culture of stromal fibroblasts, with MCF7 cancer cells (Supplementary Table 1). This is consistent with our previous studies employing the vital fluorescent dye MitoTracker, showing that mitochondrial mass is dramatically increased during MCF7-fibroblasts co-cultures [21,22]. This may also have implications for drug sensitivity to mitochondrial inhibitors, especially those targeting mitochondrial biogenesis.

**Figure 14:**
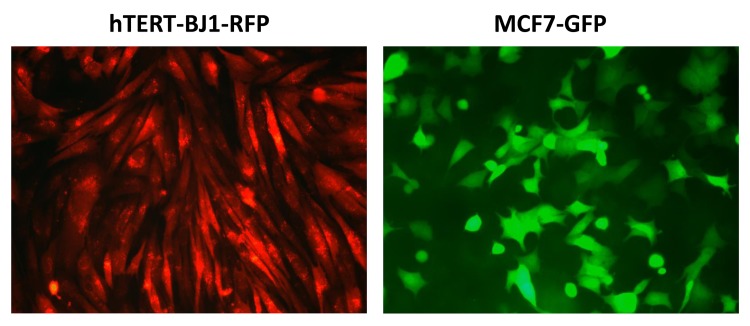
**Representative images of hTERT‐fibroblasts and MCF7 cells expressing fluorescent proteins.** hTERT‐BJ1‐fibroblasts expressing RFP and MCF7 cells expressing GFP were generated by lentiviral transduction. RFP, red fluorescent protein; GFP, green fluorescent protein.

To directly test this hypothesis, we developed an assay system to monitor the sensitivity of cancer cells to drug treatment, in the presence or absence of fibroblasts. For this purpose, we generated hTERT-BJ1-fibroblasts expressing RFP (red fluorescent protein) and MCF7 cells expressing GFP (green fluorescent protein). Representative images of these mono-cultures are shown in Figure 14.

As positive controls for this assay system, we employed two FDA-approved antibiotics (Doxycycline and Azithromycin), which have been shown to act as inhibitors of mitochondrial biogenesis, because of the long-standing evolutionary relationship between mitochondria and bacteria [8,9]. Doxycycline and Azithromycin both inhibit mitochondrial protein translation, as off-target “side-effects”, by preferentially affecting the mitochondrial ribosome.

**Figure 15:**
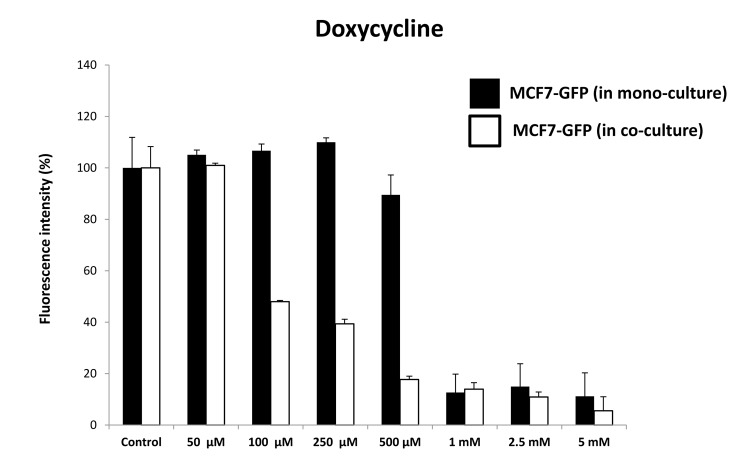
**Doxycycline preferentially targets MCF7‐GFP cells, during co‐culture with fibroblasts: Bar graphs.** Note that mono‐cultures of MCF7‐GFP cells are quantitatively more resistant to the killing effects of Doxycycline, as the concentration of Doxycycline is progressively increased, from 50 μM to 5 mM. Note that at 500 μM Doxycycline, MCF7‐GFP cells in co‐culture are ~5‐fold more sensitive, than those in mono‐cultures.

As predicted, Doxycycline preferentially targeted MCF7-GFP cells, during their co-culture with fibro- blasts, as directly compared with MCF-GFP mono- cultures (Figure 15). Quantitation of cellular GFP fluorescence was performed using a plate-reader, at the appropriate wavelength (See Materials and Methods). At 500 μM Doxycycline, MCF7-GFP cells in co- culture were ~5-fold more sensitive, than those in mono-cultures. However, the increased sensitivity of MCF7-GFP cells in co-culture was first observed at 100 μM, but not at 50 μM. Doxycycline also begins to inhibit overall protein synthesis in mammalian cells, in the range of 100 μM to 1 mM [29]. However, this effect is likely secondary to mitochondrial ATP- depletion (IC-50 = 50 μM) [30]. Therefore, Doxycycline may effectively target both “mito-stemness” and “ribo-stemness” features, by inhibiting both i) mitochondrial protein synthesis and ii) overall protein synthesis.

**Figure 16:**
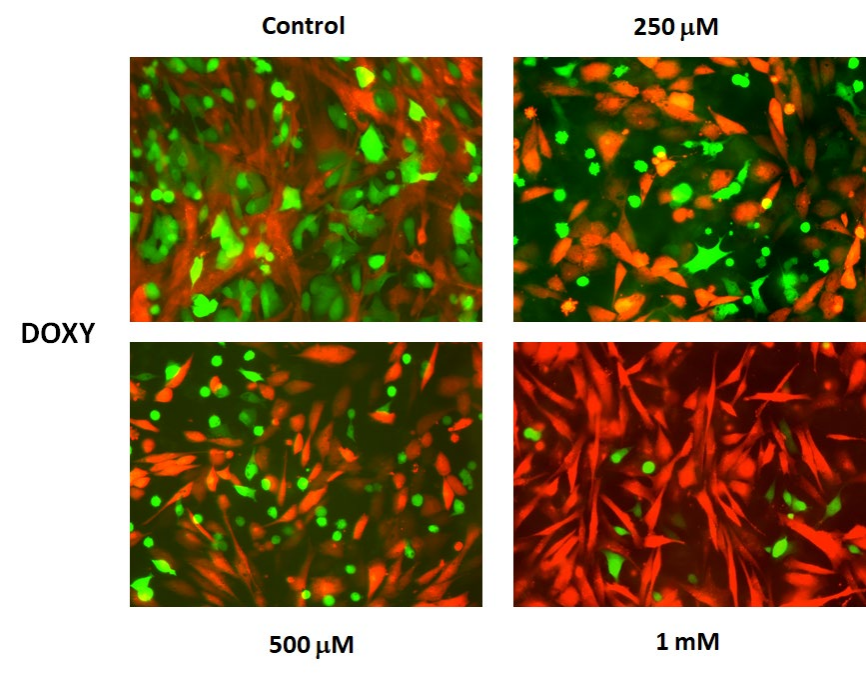
**Doxycycline preferentially targets MCF7‐GFP cells, during co‐culture with fibroblasts: Fluorescence micrographs.** Note that as the concentration of Doxycycline is progressively increased, from 250 μM to 1 mM, the green fluorescent signal is decreased.

Representative images of these MCF7-fibroblast co- cultures and their differential sensitivity to Doxycycline are shown in Figure 16. Note the progressive reductions in GFP-fluorescence.

**Figure 17:**
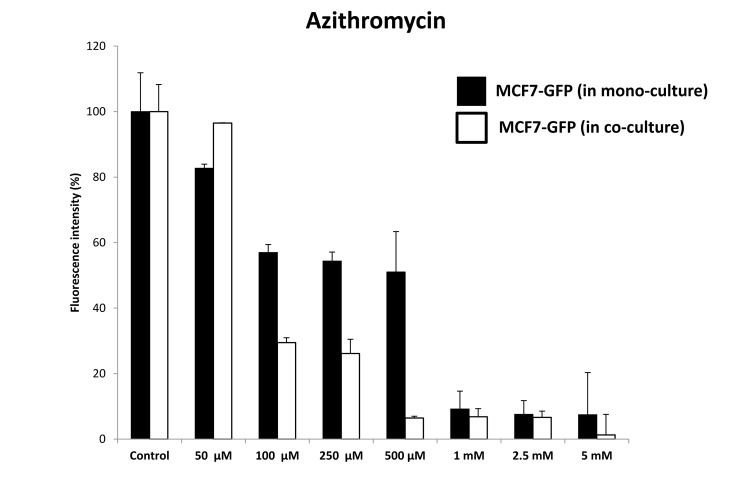
**Azithromycin preferentially targets MCF7‐GFP cells, during co‐culture with fibroblasts: Bar graphs.** Note that mono‐cultures of MCF7‐GFP cells are quantitatively more resistant to the killing effects of Azithromycin, as the concentration of Azithromycin is progressively increased, from 50 μM to 5 mM. Note that at 500 μM Azithromycin, MCF7‐GFP cells in co‐culture are ~8‐fold more sensitive, than those in mono‐cultures.

Quantitatively similar results were obtained with Azithromycin, another well-established inhibitor of mitochondrial biogenesis (Figure 17). Azithromycin preferentially targeted MCF7-GFP cells, in co-culture with fibroblasts. At 500 μM Azithromycin, MCF7-GFP cells in co-culture were ~8-fold more sensitive, than those in mono-cultures.

These pharmacological data (representing enhanced sensitivity to mitochondrial protein translation inhibitors) are consistent with the increases in mitochondrial biogenesis that we observed by proteomics analysis in MCF7-fibroblast co-cultures.

## DISCUSSION

Here, we have used MCF7-fibroblast co-cultures as a model system to dissect the molecular basis of Tamoxifen-resistance. Previously, we showed that MCF7-fibroblasts co-cultures are protected against apoptosis induced by hormonal therapies, such as Tamoxifen and Fulvestrant [16], and that this resistance could be reversed by using Metformin, a known mitochondrial inhibitor [16]. Metformin acts as an OXPHOS inhibitor and activates AMP-kinase, by functionally inactivating mitochondrial Complex I activity. However, the molecular targets that confer resistance to hormonal therapy in this epithelial-stromal model have remained elusive, although it was noted that mitochondrial mass was increased in MCF7 cells, as seen by vital staining with the probe MitoTracker [21,22].

**Figure 18:**
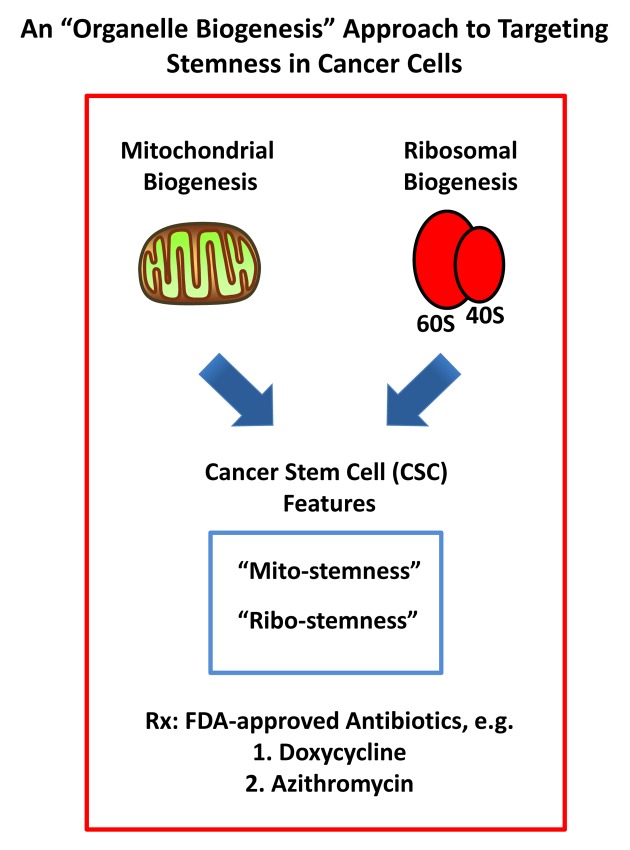
**An “organelle biogenesis” approach to cancer therapy.** A schematic diagram illustrating that breast cancer cells in co-culture increase their “mito-stemness” and “ribo-stemness” features is shown. Note the increases in mitochondrial biogenesis and the protein synthesis machinery, as enumerated in Supplementary Tables 1 to 4 and independently confirmed by Ingenuity Pathway Analysis (IPA). These findings predict increased susceptibility to treatment with FDA-approved antibiotics, such as Doxycycline and Azithromycin.

To identify potential new therapeutic targets, we subjected this co-culture system to unbiased label-free proteomics analysis. Using this approach, we observed the induction of both “mito-stemness” and “ribo- stemness” features, consistent with the induction of a more stem-like phenotype (Figure 18). In further support of this notion, the epithelial CSC marker KRT19 was induced nearly 6-fold. KRT19 is currently used clinically to identify metastatic breast cancer cells in sentinel lymph-node biopsies [24,25].

The specific candidate targets identified by proteomics analysis were then intersected with a wealth of publically-available clinical data, first to validate their expression in human breast cancer cells *in vivo*. These clinical samples were subjected to laser-capture micro- dissection to separate human breast cancer cells, from adjacent tumor stroma, before they were subjected to mRNA-based transcriptional profiling.

Unfortunately, we cannot rule out that some of the protein products that we observed were up-regulated in the MCF7-fibroblast co-cultures, were simply due to cancer cell proliferation. Nevertheless, these candidates were further intersected with transcriptional profiling data derived from human breast cancer samples, providing independent cell-type specific validation for their clinical relevance. Therefore, this approach provides an effective workflow to generate new candidate targets and a means for their clinical validation.

To assess whether these candidate biomarkers could be used as companion diagnostics for Tamoxifen- resistance, we used the targets identified by proteomics to construct a mitochondrial gene signature. Then, using publically-available clinical outcome data, we stringently evaluated the prognostic value of this Mito- Signature, which effectively predicted tumor recurrence and distant metastasis in breast cancer patients that were treated with Tamoxifen or hormonal therapy. The development of recurrence or metastasis during Tamoxifen treatment is a clear sign of treatment failure and is an accepted clinical hallmark of Tamoxifen- resistance.

We next assessed a possible treatment strategy to selectively sensitize MCF7 cells in co-culture, based on their apparent increase in mitochondrial biogenesis. For this purpose, we devised a co-culture system employing fluorescently-labeled MCF7 cells and fibroblasts. In this system, MCF7 cells expressing GFP were co- cultured with hTERT-BJ1 fibroblasts expressing RFP, so that these two cellular components were easily distinguishable.

We hypothesized that certain FDA-approved antibiotics that target host protein synthesis and mitochondrial protein translation as off-target side effects might be able to sensitize MCF7 cells, under co-culture conditions. Interestingly, our results indicate that MCF7 cells in co-culture were ~5-fold more sensitive to the effects of Doxycycline and ~8-fold more sensitive to the effects of Azithromycin. Therefore, the targeting of mitochondrial protein translation via drug repurposing may be an effective anti-cancer strategy, especially under these more physiologically-relevant culture conditions, which includes a stromal tumor-micro-environment.

Other recent studies using mono-cultures of MCF7 cells have also suggested a role for increased mitochondrial OXPHOS, in conferring Tamoxifen-resistance. For example, Tamoxifen-resistant MCF7 cells, derived from long-term cell culture with increasing concentrations of Tamoxifen, showed an increase in mitochondrial mass and mitochondrial-dependent ATP production [31]. Similarly, over-expression of a somatic mutation of the estrogen receptor (ESR1-Y537S) normally associated with the clinical development of Tamoxifen-resistance in patients, also increased mitochondrial mass and mitochondrial-dependent ATP production [32]. There- fore, three independent models of Tamoxifen-resistance mechanistically appear to show the same or a very similar mitochondrial phenotype.

As such, this highly-suggestive data implies that mitochondrial inhibitors should be tested clinically as a possible therapeutic option for the prevention and/or treatment of Tamoxifen-resistance or perhaps other forms of hormonal therapy resistance.

In this context, it is interesting to note that one of the recognized off-target side effects of Tamoxifen is that it behaves as a *bonafide* inhibitor of mitochondrial complex III and complex IV [33], while simultaneously inducing oxidative stress. Therefore, Tamoxifen treatment itself may lead to drug resistance, simply by stimulating mitochondrial biogenesis in response to its own intrinsic anti-mitochondrial activity, rather than via its direct or targeted effects on ER-alpha signaling. It is interesting to also consider these anti-mitochondrial effects of Tamoxifen, given the proposed metabolic etiology of breast cancer pathogenesis, related to mitochondrial biogenesis [34,35].

In summary, we conclude that the observed increases in “organelle biogenesis” (i.e., mitochondria and ribosomes) may represent new metabolic hallmarks of a more aggressive cancer cell phenotype. We propose that these findings can be exploited to design more broadly- applicable therapeutics and predictive companion diagnostics, to target stemness features in multiple cancer types. Our results highlight the potential clinical utility of this “organelle biogenesis” approach to cancer therapy (Figure 18).

## MATERIALS AND METHODS

### Cell culture

Cell culture experiments were carried out using human skin fibroblasts immortalized with the human telomerase reverse transcriptase (hTERT-BJ1 cells) and human MCF7 breast cancer cells. hTERT-BJ1 fibroblasts and MCF7 cells were maintained in complete media: DMEM (D6546, Sigma) supplemented with 10% fetal bovine serum (F7524, Sigma), 100 units/ml of penicillin, 100 μg/ml, streptomycin (P0781, Sigma) and 1% Glutamax (#35050087, Life Technologies). For all experiments, cells were incubated at 37°C in a humidified atmosphere containing 5% CO2.

### Co-culture versus mixed cell populations

MCF7 cells and fibroblasts were co-cultured in the presence of Nu-Serum, essentially as we previously described [16–18]. Two million MCF7 cells were co-cultured in a regular 15 cm dish with two million hTERT-BJ1 fibroblasts, seeded in a 1:1 ratio. Likewise, the same numbers of either MCF7 cells or hTERT-BJ1 fibroblasts were seeded separately in 15 cm dishes as monocultures. After 72h of cell culture cells were lysed in RIPA lysis buffer (R0278, Sigma) containing proteinase inhibitors (05 892 970 001, Roche) and kept at 4° C for 20 minutes with rotation. Lysates were cleared by centrifugation for 10 minutes at 10,000 x g and supernatants were collected. The protein concentration of the lysates was determined by using the BCA protein assay kit (23225, Pierce). Briefly, four µg of MCF7- hTERT-BJ1 co-culture lysate or two µg of hTERT-BJ1 monoculture lysate mixed with 2 µg of MCF7 monoculture protein lysate (4 µg of protein in total) were submitted to the CRUK Proteomics Core Facility, for label-free proteomic analysis. Proteomics and statistical analyses were carried out on a fee-for-service basis by Dr. Duncan Smith and his colleagues, at the Proteomics Core Facility at the Cancer Research UK Manchester Institute, University of Manchester.

### Label-free proteomics analysis

#### Chemicals and sample preparation

Formic acid, trifluoroacetic acid, ammonium formate (10 M), ammonium bicarbonate TCEP (Tris (2- carboxyethyl)phosphine hydrochloride), MMTS (Methyl methanethiosulfonate) and trypsin were all obtained from Sigma. HPLC gradient grade acetonitrile was obtained from Fisher Scientific.

#### Protein digestion

Lysate samples were thawed to room temperature and their concentrations equalised to 1 μg/μL (50 μL volume) with RIPA buffer, and further processed for trypsin digestion by sequential reduction of disulphide bonds with TCEP and alkylation with MMTS. Briefly, 1 μL benzonase (Novagen) was added to the 50 μL aliquot and placed on ice for 15 minutes. The sample was then taken to dryness using a SpeedVac, and resuspended in 22.5 μL trypsin reaction buffer (40 mM ammonium bicarbonate and 9% acetonitrile). One μL of 50 mM TCEP solution was added to each sample, mixed briefly and placed on a heater block at 60°C for 60 minutes. After cooling to room temperature, 0.5 μL of 200 mM MMTS solution was added to each sample and allowed to react for 15 minutes. Trypsin was added in two waves to ensure efficient digestion of the sample. Firstly, 20 μg of sequencing grade trypsin was resus- pended in 1800 μL of trypsin reaction buffer; 225 μL of this solution were added to each sample for digestion, and the reactions were left at 37°C overnight with shaking (600 rpm). The following morning, a further aliquot of trypsin was added. Two ml of trypsin reaction buffer was added to 20 μL of sequencing grade trypsin; 250 μL of this solution were added to each of the digest samples from overnight, and the reactions were left at 37°C for 4 hours with shaking (600 rpm). Thirty-five μL 10% formic acid were added to the 500 μL digest sample (0.7% final concentration of formic acid) to stop the digestion. The digested solution was diluted in 7.5 mL of acetonitrile containing 0.3% formic acid.

#### HILIC solid phase extraction (SPE) of peptides

PolyhydroxyethylA SPE 12 μm, 300A, 300 mg cartridges (obtained from PolyLC) were used for the HILIC procedure. Prior to use, cartridges required an overnight soak in 50 mM formic acid followed by rinsing with water the following day. Cartridges were preconditioned with 2 mL of Buffer A (90% acetonitrile, 5 mM ammonium formate, pH 2.7) fol- lowed by 2 mL of Buffer B (5 mM ammonium formate, pH 2.7) and finally re-equilibrated with 10 mL Buffer A. The diluted samples were loaded onto the cartridges and washed with a further 10 mL Buffer A. Finally, peptides were eluted in 1 mL Buffer C (9 parts Buffer B plus 1 part Buffer A) and the samples dried on a Speedvac to remove organic solvent prior to LCMS/MS analysis.

#### LC-MS/MS analysis

Lyophilized digests were resuspended in 50 μL of 0.1% TFA to give an approximate concentration of 1 μg/μL. One μL injection volumes were used throughout resulting in an on-column peptide loading of approximately 1 μg per injection. Analysis was per- formed in quintuplicate for each sample type. All LC- MS/MS analyses were performed on an LTQ Orbitrap XL mass spectrometer coupled to an Ultimate 3000 RSLCnano system (Thermo Scientific). One μL injection volumes were used throughout and samples loaded directly onto the analytical column, PepMap RSLC C18, 2 μm × 75 μm id × 50 cm (Thermo Scientific). The composition (v/v) of LC buffers were as follows; Buffer A - 99.9% water plus 0.1% formic acid and Buffer B - 80% acetonitrile, 19.9% water and 0.1% formic acid. Peptides were loaded directly onto the column at a flow rate of 400 nl/min with an initial mobile phase composition of 1% B. The organic strength was increased linearly from 1% to 22.5% B over 22.5 minutes again at 400 nl/min, followed by an increase to 24.8% B over the next 2.6 minutes with a concomitant reduction in flow rate to 300 nl/min, and to 39% B over a further 14 minutes. A further increase to 60% B over the next 5 minutes was followed by a ramp to 95% B over 2.5 minutes where it was held for a further 2 minutes. The column was then allowed to re- equilibrate to 1% B for a total analysis time of 74 minutes. The mass spectrometer was instructed to perform data dependent acquisition on the top six precursor ions, which were measured in the Orbitrap FTMS detector over the mass range 370–1200 m/z, at a nominal resolution of 60,000. MS/MS spectra were acquired in the ion trap under CID conditions with normalized collision energy of 35, isolation width of 3 Th, Q value of 0.25 and 30 ms activation time. Gas- phase fractionation was performed on the five replicate injections such that MS/MS data was collected for precursor ion range 370–494 m/z Injection 1, 494–595 m/z Injection 2, 595–685 m/z Injection 3, 685–817 m/z Injection 4 and 817–1200 m/z Injection 5.

#### Statistical analysis

Xcalibur raw data files acquired on the LTQ-Orbitrap XL were directly imported into Progenesis LCMS software (Waters Corp) for peak detection and alignment. Data were analysed using the Mascot search engine. Five replicates were analysed for each sample type. Statistical analyses were performed using ANOVA and only fold-changes in proteins with a p- value less than 0.05 were considered significant.

#### Ingenuity pathway analyses

Pathway and function analyses were generated using Ingenuity Pathway Analysis (IPA) (Ingenuity systems, http://www.ingenuity.com), which assists with proteomics data interpretation via grouping differentially expressed genes or proteins into known functions and pathways. Pathways with a z score > 1.9 were considered as significantly activated, and pathways with a z score < −1.9 were considered as significantly inhibited.

### Validation with transcriptional profiling of breast cancer patient samples

To directly establish the clinical relevance of our findings from proteomics analysis of MCF7-fibroblast co-cultures, we re-analyzed the publically-available transcriptional profiles [26] of epithelial breast cancer cells and adjacent tumor stromal cells that were physically separated by laser-capture microdissection (from N=28 human breast cancer patients).

### Validation with Kaplan-Meier (K-M) analyses of breast cancer patient samples

To perform K-M analysis of mitochondrial gene transcripts, we used an open-access online survival analysis tool to interrogate publically available microarray data from up to 3,455 breast cancer patients [27]. This allowed us to determine their prognostic value [28]. For this purpose, we primarily analyzed data from ER(+) patients that were LN(+) at diagnosis and were of the luminal A sub-type, that were primarily treated with Tamoxifen and not other chemotherapy (N=152 patients). In this group, 100% the patients received some form of hormonal therapy and ~95% of them received Tamoxifen. Biased and outlier array data were excluded from the analysis. This allowed us to identify mitochondrial gene transcripts, with significant prognostic value. Hazard-ratios were calculated, at the best auto-selected cut-off, and p- values were calculated using the logrank test and plotted in R. K-M curves were also generated online using the K-M-plotter (as high-resolution TIFF files), using univariate analysis:

http://kmplot.com/analysis/index.php?p=service&cancer=breast

This allowed us to directly perform in silico validation of these mitochondrial biomarker candidates. The multi- gene classifier function of the program was used to test the prognostic value of short mitochondrial gene signatures, using the mean expression of the selected probes.

### Validation via drug treatment of fluorescently- labeled MCF7-fibroblast co-cultures

hTERT-BJ1-RFP cells and MCF7-GFP cells were first generated by stable transduction with lenti-viral vectors. Then, these cells were plated into clear flat bottom 96- well black microplates (Corning; CLS3603**).** Mono- cultures were generated by plating either hTERT-BJ1- RFP (8,000 cells/well) or MCF7-GFP cells (8,000 cells/well). In parallel, co-cultures were generated by co-plating hTERT-BJ1-RFP (6,000 cells/well) with MCF7-GFP (2,000 cells/well). The next day, the plates were treated with either Doxycycline or Azithromycin (or vehicle controls) and were incubated for 72 hours at 37^o^C. The cell culture plates were then read with a plate-reader at 545/590 nm (for RFP) and at 490/510 nm (for GFP). Background readings were subtracted from the fluorescent signal and data were then normalized to controls.

## SUPPLEMENTARY MATERIAL

Supplementary Tables
