# Supplementary material for: Mitochondrial and ribosomal biogenesis are new hallmarks of stemness, oncometabolism and biomass accumulation in cancer: Mito‐stemness and ribo‐stemness features
Source: Aging (Albany NY). 2019 Jul 16;11(14):4801–35. doi: 10.18632/aging.102054 (PMC6682537; doi:10.18632/aging.102054)
Supplement: Supplementary Tables [file aging-11-102054-s001.pdf]

SUPPLEMENTARY MATERIAL

Supplementary Tables

Supplementary Table 1. Proteomics Analysis: Mitochondrial-Related Proteins Up-regulated in MCF7-Fibroblast Co-Cultures.

Gene Symbol	Description	Fold-Increase	Mito-Complex	MRP/Chaperone
NDUFAF2	Mimitin, c-Myc-induced mitochondrial protein; B17.2L (related to NDUFAF4/**HRPAP20**)	Infinity	I	Chaperone
GPD2	Glycerol-3-phosphate dehydrogenase, mitochondrial (ROS/H2O2 Production)	238.91		
AIFM1	Apoptosis-inducing factor 1, mitochondrial (NADH oxidase activity)	237.74	I, III, IV	Chaperone
PRKDC	DNA-dependent protein kinase catalytic subunit (mitochondrial genome maintenance)	102.78		
DNAJA3	HSP40, DnaJ homolog, subfamily A, member 3, mitochondrial	76.43		Chaperone
MRPL43	Mitochondrial ribosomal protein L43	66.38		MRP
COX4I1	Cytochrome c oxidase subunit 4 isoform 1	56.68	IV	
HSPD1	HSP60, 60 kDa heat shock protein, mitochondrial	29.29		Chaperone
MRRF	Ribosome-recycling factor, mitochondrial	23.53		MRP
SLC25A5	Solute Carrier Family 25 (Mitochondrial Carrier; ADP/ATP Translocator), Member 5	14.49		
VDAC2	Voltage-dependent anion-selective channel protein 2	13.60		
UQCRFS1	RISP, Ubiquinol-cytochrome C reductase iron-sulfur subunit, (Rieske iron-sulfur protein)	13.07	III	
VDAC1	Voltage-dependent anion-selective channel protein 1	10.05		
ECH1	Delta(3,5)-Delta(2,4)-dienoyl-CoA isomerase, mitochondrial	8.51		
MCCC2	Methylcrotonoyl-CoA carboxylase beta chain, mitochondrial	4.52		
MRPS28	MRPS28 protein	3.86		MRP
ATP5A1	ATP synthase subunit alpha, mitochondrial (EC 3.6.3.14)	3.83	V	
SUCLG2	Succinyl-CoA ligase [GDP-forming] subunit beta, mitochondrial	3.58		
ABAT	4-aminobutyrate aminotransferase, mitochondrial	3.44		
IMMT	Mitochondrial inner membrane protein	3.27		
AK4	Adenylate kinase isoenzyme 4, mitochondrial	3.02		
GOT2	Aspartate aminotransferase, mitochondrial	2.87		
HSPA9	heat shock 70kDa protein 9 (mortalin), mitochondrial	2.47		Chaperone
TUFM	Elongation factor Tu, mitochondrial	2.37		
CLUH	Clustered mitochondria protein homolog	2.15		
OAT	Ornithine aminotransferase, mitochondrial	2.14		
IDH1	Isocitrate dehydrogenase [NADP], cytoplasmic	2.10		
SLC25A3	Solute carrier family 25 (mitochondrial carrier; phosphate carrier), member 3	2.09		
NDUFA5	NADH dehydrogenase [ubiquinone] 1 alpha subcomplex subunit 5	2.08	I	
UQCRC2	Cytochrome b-c1 complex subunit 2, mitochondrial	2.07	III	
ETFA	Electron transfer flavoprotein subunit alpha, mitochondrial	2.05		
PPA2	Inorganic pyrophosphatase 2, mitochondrial	2.03		
ECHS1	Enoyl-CoA hydratase, mitochondrial	2.02		
PPT1	Palmitoyl-protein thioesterase 1	1.91		

DLD	Dihydrolipoyl dehydrogenase, mitochondrial	1.89		
CHCHD3	Coiled-coil-helix-coiled-coil-helix domain-containing protein 3, mitochondrial	1.89		
ATP5H	ATP synthase subunit d, mitochondrial	1.88	V	
IARS2	Isoleucine--tRNA ligase, mitochondrial	1.86		
LRPPRC	Leucine-rich PPR-motif containing, mitochondrial	1.85		
PC	Pyruvate carboxylase, mitochondrial	1.79		
HADHA	Trifunctional enzyme subunit alpha, mitochondrial	1.74		
SLC25A13	Calcium-binding mitochondrial carrier protein Aralar2	1.74		
TRAP1	Heat shock protein 75 kDa, mitochondrial	1.72		Chaperone
ACADSB	Short/branched chain specific acyl-CoA dehydrogenase, mitochondrial	1.72		
CPT1A	Carnitine palmitoyltransferase 1A, mitochondrial	1.65		

-Proteins listed above (45 in total) were all upregulated in MCF7-Fibroblast Co-cultures ($p < 0.05$). **MRP**, mitochondrial ribosomal proteins

Supplementary Table 2. Proteomics Analysis: Ribosomal Proteins Up-regulated in MCF7-Fibroblast Co-Cultures.

Gene Symbol	Description	Fold-Increase
RPL4	60S ribosomal protein L4	Infinity
RPL15	60S ribosomal protein L15	2,238.12
RPL19	60S ribosomal protein L19	168.84
RPL36AL	60S ribosomal protein L36a-like	54.47
RPL24	60S ribosomal protein L24	43.56
RPL10	60S ribosomal protein L10	42.78
RPL29	60S ribosomal protein L29	20.53
RPL18A	60S ribosomal protein L18a	17.08
RPL8	60S ribosomal protein L8	5.74
RPL34	60S ribosomal protein L34	5.36
RPL13	60S ribosomal protein L13	5.31
RPL14	60S ribosomal protein L14	3.09
RPL3	60S ribosomal protein L3	3.04
RPL27a	60S ribosomal protein L27a	2.99
RPLP2	60S acidic ribosomal protein P2	2.90
RPL6	60S ribosomal protein L6	2.05
RPL5	60S ribosomal protein L5	2.04
RPL28	60S ribosomal protein L28	2.00
RPS29	40S ribosomal protein S29	Infinity
RPS10	40S ribosomal protein S10	74.47
RPS6	40S ribosomal protein S6	23.37
RPS18	40S ribosomal protein S18	9.20
RPS27A	40S ribosomal protein S27a	5.19
RPS2	40S ribosomal protein S2	2.98
RPS27A	40S ribosomal protein S27a	2.96
RPS3A	40S ribosomal protein S3A	2.39
RPS19	40S ribosomal protein S19	2.05
RPS11	40S ribosomal protein S11	2.00

-Proteins listed above (28 in total) were all upregulated in MCF7-Fibroblast Co-cultures ($p < 0.05$).

Supplementary Table 3. Proteomics Analysis: Other Chaperones Up-regulated in MCF7-Fibroblast Co-Cultures.

Gene Symbol	Description	Fold-Increase
HSP90AB1	Heat Shock Protein 90kDa Alpha (Cytosolic), Class B Member 1	Infinity
HSPA5	78 kDa glucose-regulated protein	82.18
HSP90AA1	Heat Shock Protein 90kDa Alpha (Cytosolic), Class A Member 1	79.52
HSPB1	Heat Shock 27kDa Protein 1	35.73
HSPA8	Heat shock cognate 71 kDa protein	8.42
HSPA4	Heat shock 70 kDa protein	5.38
STIP1	Stress-induced-phosphoprotein 1 (HSP70/HSP90-organizing protein)	2.99
CDC37	HSP90 co-chaperone Cdc37	2.83
HSPA2	Heat shock-related 70 kDa protein 2	2.59
HSPH1	Heat shock protein 105 kDa	2.53

-Proteins listed above (10 in total) were all upregulated in MCF7-Fibroblast Co-cultures ($p < 0.05$).

Supplementary Table 4. Proteomics Analysis: Proteins Involved in mRNA Translation Initiation, Polypeptide Elongation, tRNA Synthesis and Amino Acid Uptake are All Up-regulated in MCF7-Fibroblast Co-Cultures.

Gene Symbol	Description	Fold-Increase
**Translation initiation factors (required for mRNA binding to ribosomes)**		
EIF2S1	Eukaryotic translation initiation factor 2 subunit 1	291.47
EIF3H	Eukaryotic translation initiation factor 3 subunit H	81.05
EIF4H	Eukaryotic translation initiation factor 4H	31.21
EIF4A1	Eukaryotic translation initiation factor 4A-I	18.85
EIF5	Eukaryotic translation initiation factor 5	8.72
EIF3A	Eukaryotic translation initiation factor 3 subunit A	2.94
EIF2S3L	Putative eukaryotic translation initiation factor 2 subunit 3-like protein	2.86
EIF3E	Eukaryotic translation initiation factor 3 subunit E	2.30
EIF3B	Eukaryotic translation initiation factor 3 subunit B	1.85
**Elongation factors (promote delivery of aminoacyl tRNAs to the ribosome)**		
EEF2	Elongation factor 2	18.96
EEF1G	Elongation factor 1-gamma	2.45
TUFM	Elongation factor Tu, mitochondrial	2.37
EEF1A2	Elongation factor 1-alpha 2	2.36
EEF1A1	Elongation factor 1-alpha	2.03
EEF1D	Elongation factor 1-delta	1.86
**Enzymes for tRNA Synthesis**		
QARS	Glutamine--tRNA ligase, cytoplasmic	31.90
DARS	Aspartate--tRNA ligase, cytoplasmic	9.69
MARS	Methionine-tRNA synthetase, cytoplasmic	3.44
RTCB	tRNA-splicing ligase RtcB homolog (C22orf28)	3.25
DTD1	D-tyrosyl-tRNA(Tyr) deacylase 1	3.10
YARS	Tyrosine--tRNA ligase, cytoplasmic	2.38
RARS	Arginine--tRNA ligase, cytoplasmic	2.23
AARS	Alanyl-tRNA synthetase, cytoplasmic	2.23
IARS2	Isoleucine--tRNA ligase, mitochondrial	1.86
AIMP2	Aminoacyl tRNA synthase complex-interacting multifunctional protein 2	1.70
GARS	Glycine--tRNA ligase, cytoplasmic	1.70
EPRS	Bifunctional glutamate/proline--tRNA ligase	1.67
TRMT112	tRNA methyltransferase 112 homolog	1.66
**Amino Acid Transporters**		
SLC6A19	Neutral amino acid transporter B(0)	4.18
SLC7A5	Solute carrier family 7 (Cationic AA transporter, y+ system), member 5	3.05

-Proteins listed above (30 in total) were all upregulated in MCF7-Fibroblast Co-cultures ($p < 0.05$).

Supplementary Table 5. Proteomics Analysis: Markers of Cell Proliferation and Stemness are Up-regulated in MCF7-Fibroblast Co-Cultures.

Gene Symbol	Description	Fold-Increase	Specificity
MKI67	Antigen KI-67	4,531.38	Expressed in all cycling cells, except for resting cells in the G0-phase; Associated also with ribosomal RNA (rRNA) synthesis and, thus, protein synthesis
KRT19	Keratin, type I cytoskeletal 19	5.94	Marker of CSCs and breast cancer metastasis (sentinel lymph node)
PCNA	Proliferating cell nuclear antigen	3.87	Marker for the G1/S phase of the cell cycle

-Proteins listed above were upregulated in MCF7-Fibroblast Co-cultures ($p < 0.05$).
-In human breast cancer cells *in vivo*, KRT19 was transcriptionally up-regulated by 4.39-fold (p=2.66E-05), relative to the adjacent tumor stroma. Similarly, PCNA levels were up-regulated by 3.58-fold (p=3.64E-04).

Supplementary Table 6. Nuclear Mitochondrial Gene Transcripts Up-regulated in Human Breast Cancers (Cancer Epithelia vs. Tumor Stroma).

Gene Symbol	Fold-Increase	P-Value
UQCRFS1	5.71	2.45E-07
MCCC2	5.48	5.78E-07
ATP5A1	5.01	3.09E-06
UQCRC2	4.84	5.73E-06
IMMT	4.71	8.89E-06
IARS2	4.70	9.15E-06
GOT2	4.58	1.40E-05
LRPPRC	4.34	3.15E-05
ECHS1	4.05	8.22E-05
ATP5H	4.01	9.48E-05
VDAC2	3.99	0.0001
DLD	3.78	0.0002
PPT1	3.76	0.0002
SLC25A3	3.76	0.0002
HSPA9	3.69	0.0002
SLC25A5	3.49	0.0005
HSPD1	3.42	0.0006
COX4I1	3.39	0.0007
TUFM	3.38	0.0007
HADHA	3.27	0.0009
PPA2	3.19	0.001
IDH1	3.18	0.001
OAT	3.17	0.001
SUCLG2	3.03	0.002
DNAJA3	2.92	0.003
NDUFA5	2.75	0.004
CHCHD3	2.74	0.004
SLC25A13	2.69	0.005
VDAC1	2.64	0.005
MRPS28	2.25	0.01
PRKDC	2.14	0.02
ABAT	2.08	0.02
ECH1	1.97	0.03
ETFA	1.75	0.04

-Transcriptional profiling data derived from the analysis of N=28 breast cancer patients are shown, high-lighting the levels of fold-upregulation observed in the epithelial cancer cell compartment (relative to the tumor stroma), and corresponding p-values derived from the analysis of these clinical samples.

-Proteins listed above (34 in total) were all upregulated in MCF7-Fibroblast Co-cultures (Compare with **Supplementary Table 1**) ($p < 0.05$).

Supplementary Table 7. Ribosomal Protein Gene Transcripts Up-regulated in Human Breast Cancers (Cancer Epithelia vs. Tumor Stroma).

Gene Symbol	Fold-Increase	P-Value
RPL24	5.38	8.11E-07
RPL3	5.01	3.14E-06
RPL10	4.91	4.48E-06
RPL15	4.60	1.28E-05
RPL13	4.48	1.98E-05
RPL14	4.45	2.15E-05
RPL6	4.00	9.86E-05
RPL19	3.98	0.0001
RPL8	3.86	0.00015
RPLP2	3.80	0.0002
RPL34	3.63	0.0003
RPL4	3.05	0.002
RPL29	2.94	0.002
RPL27A	2.87	0.003
RPL5	2.74	0.004
RPL28	2.33	0.01
RPL18A	2.28	0.01
RPL36AL	1.70	0.048
RPS18	4.96	3.71E-06
RPS27A	4.63	1.19E-05
RPS3A	4.59	1.35E-05
RPS6	4.47	2.04E-05
RPS10	4.18	5.34E-05
RPS19	4.17	5.49E-05
RPS11	3.58	0.0004
RPS2	3.36	0.0007
RPS29	2.31	0.01

-Transcriptional profiling data derived from the analysis of N=28 breast cancer patients are shown, high-lighting the levels of fold-upregulation observed in the epithelial cancer cell compartment (relative to the tumor stroma), and corresponding p-values derived from the analysis of these clinical samples.

-Proteins listed above (27 in total) were all upregulated in MCF7-Fibroblast Co-cultures (Compare with **Supplementary Table 2**). ($p < 0.05$).

Supplementary Table 8. Other Chaperone Gene Transcripts Up-regulated in Human Breast Cancers (Cancer Epithelia vs. Tumor Stroma).

Gene Symbol	Fold-Increase	P-Value
HSP90AB1	4.93	4.03E-06
HSPA5	3.89	0.0001
HSP90AA1	3.76	0.0002
HSPA4	3.75	0.0002
HSPB1	3.27	0.001
HSPH1	3.18	0.001
HSPA8	3.11	0.002

-Transcriptional profiling data derived from the analysis of N=28 breast cancer patients are shown, high-lighting the levels of fold-upregulation observed in the epithelial cancer cell compartment (relative to the tumor stroma), and corresponding p-values derived from the analysis of these clinical samples.

-Proteins listed above (7 in total) were all upregulated in MCF7-Fibroblast Co-cultures (Compare with **Supplementary Table 3**). ($p < 0.05$).

Supplementary Table 9. Transcripts of Genes Associated with mRNA Translation Initiation, Polypeptide Elongation, and tRNA Synthesis Are All Up-regulated in Human Breast Cancers (Cancer Epithelia vs. Tumor Stroma).

Gene Symbol	Fold-Increase	P-Value
**Translation initiation factors (required for mRNA binding to ribosomes)**		
EIF4H	4.77	7.20E-06
EIF3H	4.70	9.25E-06
EIF5	3.90	0.0001
EIF3E	3.57	0.0004
EIF3A	2.51	0.008
EIF3B	2.21	0.02
**Elongation factors (promote delivery of aminoacyl tRNAs to the ribosome)**		
EEF2	4.01	9.29E-05
EEF1G	3.71	0.0002
TUFM	3.38	0.0007
EEF1A1	3.16	0.001
EEF1D	2.50	0.008
**Enzymes for tRNA Synthesis**		
IARS2	4.70	9.15E-06
RTCB	4.59	1.37E-05
MARS	4.35	3.00E-05
EPRS	4.06	8.10E-05
QARS	3.73	0.0002
DARS	3.43	0.0006
DTD1	1.78	0.04
YARS	1.72	0.046

-Transcriptional profiling data derived from the analysis of N=28 breast cancer patients are shown, high-lighting the levels of fold-upregulation observed in the epithelial cancer cell compartment (relative to the tumor stroma), and corresponding p-values derived from the analysis of these clinical samples.

-Proteins listed above (19 in total) were all upregulated in MCF7-Fibroblast Co-cultures (**Compare with Supplemental Table 4**). ($p < 0.05$).

Supplementary Table 10. Proteomics Analysis: Stromal CAF Markers are Up-regulated in MCF7-Fibroblast Co-Cultures

Gene Symbol	Description	Fold-Increase
**Glycolytic Enzymes**		
PKM	Pyruvate kinase	Infinity
LDHA	L-Lactate dehydrogenase A	426.16
PGD	6-Phosphogluconate dehydrogenase, decarboxylating	13.59
ENO1	Alpha-Enolase	11.60
ALDOA	Fructose-bisphosphate aldolase	7.14
PGI	Glucose-6-phosphate isomerase	5.02
PFK	6-Phosphofructokinase	4.14
PGK1	Phosphoglycerate kinase 1	4.38
TPI1	Triosephosphate isomerase 1	2.66
G6PD	Glucose-6-phosphate 1-dehydrogenase	2.28
**Cytoskeletal and Extracellular Matrix Proteins**		
MYH9	Myosin Heavy Chain 9	226.32
COL4A1	Collagen Type IV Alpha 1 Chain	211.46
CTTN	Cortactin	202.50
DNHD1	Dynein heavy chain domain-containing protein 1	195.80
NEFL	Neurofilament light polypeptide	124.55
FLNA	Filamin-A	60.54
KIF5C	Kinesin heavy chain isoform 5C	60.34
TLN1	Talin-1	33.25
S100P	S100 Calcium Binding Protein P	21.23
SPTAN1	Spectrin alpha chain, non-erythrocytic 1	11.78
MYH11	Myosin Heavy Chain 11	8.99
DYNC1H1	Cytoplasmic dynein 1 heavy chain 1	8.51
COL1A1	Collagen Type I Alpha 1 Chain	7.38
CFL1	Cofilin-1	6.36
MYO18B	Myosin XVIIIB	6.11
PLEC1	Plectin 1, intermediate filament binding protein	5.48
SRRM2	Serine/arginine repetitive matrix protein 2	5.18
S100A16	S100 Calcium Binding Protein A16	4.87
SRRM1	Serine/arginine repetitive matrix protein 1	4.77
ACTN2	Alpha-actinin-2	4.31
MAP4	Microtubule-associated protein	4.22
DNM2	Dynamin-2	4.10
ARPC1B	Actin related protein 2/3 complex, subunit 1B	3.43
MACF1	Microtubule-actin cross-linking factor 1	3.26
VCL	Vinculin	2.66
TPM4	Tropomyosin alpha-4	2.65
ANKRD12	Ankyrin repeat domain-containing protein 12	2.58
VIL2	Villin 2 (Ezrin)	2.57
DYNC1LI2	Cytoplasmic dynein 1 light intermediate chain 2	2.42
VIM	Vimentin	2.37
NEFM	Neurofilament medium polypeptide	2.35
TUBB3	Tubulin beta-3	2.35
**Senescence and the SASP (Senescence-Associated Secretory Phenotype)**		
HMGB2	High mobility group protein B2	64.01
HMGB21	High mobility group protein B1	4.00
**Autophagy/Lysosomal Markers**		
ATP6V1E1	ATPase, H+ transporting, lysosomal, V1 subunit E1	2.60
ATP6V1A	V-type proton ATPase catalytic subunit A	2.56
CTSD	Cathepsin D	2.08

CTSZ	Cathepsin Z	2.07

-Proteins listed above were all upregulated in MCF7-Fibroblast Co-cultures ($p < 0.05$).
